# Antibodies to Conserved Surface Polysaccharides Protect Mice Against Bacterial Conjunctivitis

**DOI:** 10.1167/iovs.18-23795

**Published:** 2018-05

**Authors:** Tanweer S. Zaidi, Tauqeer Zaidi, Gerald B. Pier

**Affiliations:** Division of Infectious Diseases, Department of Medicine, Brigham and Women's Hospital, Harvard Medical School, Boston, Massachusetts, United States

**Keywords:** conjunctivitis, PNAG, vaccine, MAb, *S. pneumoniae*, *P. aeruginosa*, MRSA

## Abstract

**Purpose:**

Bacterial conjunctivitis is a major problem in ocular health. Little is known about protective immune effectors in the conjunctiva. We evaluated whether opsonic antibody to the conserved surface/capsular polysaccharide poly-*N*-acetyl glucosamine (PNAG) expressed by *Streptococcus pneumoniae* and *Staphylococcus aureus* was protective against bacterial conjunctivitis, as well as an antibody to the *Pseudomonas aeruginosa* surface polysaccharide alginate.

**Methods:**

Bacteria were injected directly into the conjunctivae of either A/J mice or into conjunctivae of wild type C57Bl/6 mice for comparisons to responses of recombination activating gene 1-knock out (RAG 1 KO) or germ-free mice in the C57Bl/6 genetic background. Human IgG1 monoclonal antibodies (MAb) to either PNAG or alginate were administered as follows: direct injection of 10 μg into the conjunctivae or topical application onto the cornea 4, 24, and 32 hours post infection; or intraperitoneal injection of 200 μg 18 hours prior to and then 4, 24, and 32-hours postinfection. After 48 hours, eyes were scored for pathology, mice were euthanized, and CFU/conjunctiva was determined.

**Results:**

All methods of antibody administration reduced *S. pneumoniae*, *S. aureus*, or *P. aeruginosa* pathology and bacterial levels in the conjunctivae. Histopathologic analysis showed severe inflammatory cell infiltrates in conjunctivae of mice treated with control MAb, whereas immune mice showed only very mild cellular infiltration. The protective effect of MAb to PNAG was abolished in RAG 1 KO and germ-free mice.

**Conclusions:**

Antibodies to both PNAG and alginate demonstrated therapeutic efficacy in models of *S. pneumoniae*, *S. aureus*, and *P. aeruginosa* conjunctivitis, validating the protective capacity of antibodies to surface polysaccharides in distinct ocular tissues.

*Streptococcus pneumoniae*, *Staphylococcus aureus*, and *Pseudomonas aeruginosa* are important worldwide pathogens, causing a variety of systemic diseases such as pneumonia and bacteremia.^1–5^ These bacteria are also common causes of ocular infections including keratitis and conjunctivitis.^6–9^ *Streptococcus pneumoniae* is reported to be the most common bacterial pathogen of acute conjunctivitis in children.^7^ *Staphylococcus aureus*, including methicillin-resistant *S. aureus* (MRSA), along with *Pseudomonas aeruginosa*, are increasingly isolated in elderly patients with conjunctivitis.^10,11^ *Pseudomonas aeruginosa* has been noted to cause significant conjunctival disease in neonatal intensive care units.^12^ The clinical impact has recently been estimated to have an incidence of 135 cases per 10,000 individuals, leading to an economic impact or around $589 million per year in the United States alone.^13^

Many virulence factors contribute to the severity of infections caused by microbial pathogens and surface polysaccharides are among the best characterized of these.^14–19^ Poly-*N*-acetylglucosamine (PNAG) is a conserved surface polysaccharide produced by major pathogens including *S. pneumoniae* and *S. aureus*,^15^ but not *P. aeruginosa*.^20^ For a capsule, this latter organism makes a β-1-4-linked polymer of O-acetylated mannuronic acid, known as alginate.^21^ In prior studies of *S. aureus* and MRSA keratitis in mice, we found that a human IgG1 monoclonal antibody (MAb) to PNAG, given either prophylactically via intraperitoneal (IP) injection or therapeutically as a topical treatment, markedly reduced corneal pathology and bacterial burdens.^22^ We obtained comparable results using antibody to PNAG in scratch-injured murine eyes challenged with the fungal pathogens *Candida albicans*, *Fusarium solani*, or *Aspergillus flavus*.^23^ Similarly, a human IgG1 MAb to alginate is protective against experimental *P. aeruginosa* keratitis.^24,25^ However, there have not been any major evaluations of the local or systemic impact of antibody, phagocytes, and complement in the conjunctiva and whether these immune effectors can combine to prevent pathology and disease. We developed a mouse model of bacterial conjunctivitis to evaluate the protective activity of the human IgG1 MAb to PNAG. Antibody was delivered by systemic, topical, or intraconjunctival routes. Additionally, we used mice deficient in innate lymphocyte effectors to ascertain the role of these immune cells in antibody-mediated immunity. Finally, we used germ-free mice to discover if the microbiome-mediated maturation of the immune system was necessary to establish effective antibody-mediated immunity in the conjunctiva.

## Materials and Methods

### Bacterial Strains

*Streptococcus pneumoniae* strains D-39 and ATCC8 were obtained from BEI Resources (Manassas, VA, USA) and grown in Todd-Hewitt Broth (BD Difco, Houston, TX, USA) with 1% added glucose statically in a 5% CO_2_ incubator until they reached the mid-log phase of growth. *Staphylococcus aureus* LAC (a USA300 MRSA strain) was obtained from the Network on Antimicrobial Resistance in *S. aureus* (now available from BEI Resources). *Staphylococcus aureus* strains were grown overnight on tryptic soy agar, then inoculated into liquid tryptic soy broth with 1% glucose and incubated at 37°C to late log phase. Bacterial cells in growth media were centrifuged and suspended in sterile PBS, and then the concentration was adjusted using the OD_650_ nm absorbance to achieve the desired inoculum in a volume of 5 μL. *Pseudomonas aeruginosa* strains were from our collection of clinical isolates from patients with keratitis^26^ and were grown overnight at 37°C on tryptic soy agar, then cells were scraped from the plate and suspended in PBS to achieve the desired inoculum in a volume of 5 μL.

### Mouse Manipulations

All animal studies were carried out under the National Institutes of Health Guide for the Care and Use of Laboratory Animals. The Brigham and Women's Hospital Institutional Animal Care and Use Committee approved all protocols. In addition, we adhered to the ARVO Statement for the Use of Animals in Ophthalmic and Vision Research.

A/J and C57Bl/6 female mice 6 to 8 weeks old were purchased from Jackson Laboratories (Bar Harbor, ME, USA). A/J mice were used to establish basic parameters of the infection system, whereas wild type (WT) C57Bl/6 mice were used as controls for transgenic recombination activating gene 1-knock out (RAG 1 KO) mice, also purchased from Jackson Laboratories. Germ-free C57Bl/6 mice were obtained from the Harvard Digestive Diseases Center Germ Free and Gnotobiotic Microbiology Core (Boston, MA, USA).

### Antibodies to PNAG

An extensively characterized fully human IgG1 MAb to PNAG (MAb F598) was used to provide immunity to *S. pneumoniae* and *S. aureus*.^27,28^ Controls received the human IgG1 MAb F429 specific to *P. aeruginosa* alginate. Importantly, the constant regions of the heavy and light chains of these two MAbs are identical.^29^ In experiments using *P. aeruginosa* to cause conjunctivitis, the roles of the MAbs were reversed, with the MAb to PNAG serving as the control and the MAb to alginate serving as the test material.

### Animal Model of Conjunctivitis

To induce conjunctival infection, *S. pneumoniae*, *S. aureus*, or *P. aeruginosa* (∼10^7^ CFU/conjunctiva) were injected directly into the conjunctiva of mice in a 5 μL volume. For prophylactic plus therapeutic immunotherapy experiments, 18 hours prior to infection and 4, 24, and 32 hours postinfection, 200 μg of the human IgG1 MAb to PNAG, MAb F598, or control IgG1 MAb F429 were IP injected. For local immunotherapeutic treatments, 10 μg of MAbs were either directly injected in 5 μL volumes into the conjunctivae or topically applied onto the cornea at 4, 24, and 32 hours postinfection. These doses were chosen as they are consistent with those successfully used previously for immunotherapy of keratitis in mice^23^ and represent a low dose of antibody that would minimize the cost of such agents if they were effective in this setting. Pathological changes in the conjunctiva were assessed daily by an investigator unaware of the treatment received by the mice by using a 0 to 4 scale. The palpebral and bulbar conjunctivae were scored for erythema, edema, and exudation. The scoring system used was the following: 0, no change from contralateral control conjunctiva; 1, slight erythema or edema of either the palpebral or bulbar conjunctiva; 2, definite erythema or edema of either the palpebral or bulbar conjunctiva; 3, definite erythema or edema of both the palpebral and bulbar conjunctivae; and 4, definite erythema or edema of both the palpebral and bulbar conjunctivae and the presence of exudate.

After 48 hours, animals were euthanized, eyes enucleated, and CFU/conjunctiva determined by homogenization, serial dilutions, and plating for bacterial enumeration.

### Histopathology Examinations

Infected eyes from mice treated with MAb to PNAG or the control antibody were obtained for histopathologic analysis by enucleating eyes from euthanized mice and fixing them in 4% formaldehyde, followed by embedding in paraffin. Four-μm sections were cut and stained with hematoxylin-eosin to visualize tissue morphology and cellular infiltrates.

### Analysis of PMN Infiltration Into Corneas

After 48 hours of conjunctival infection, eyes were removed from euthanized mice and homogenized in PBS containing 0.5% hexadecyltrimethylammonium bromide in a protease inhibitor cocktail. Samples were freeze-thawed three times, sonicated on ice, then centrifuged at 14,000*g* for 10 minutes at 4°C. Myeloperoxidase (MPO) levels were evaluated in homogenates by using an ELISA kit (Abcam, Cambridge, MA, USA) following the instructions in the user's manual.

### Statistical Analysis

For pair-wise comparisons, the nonparametric Mann-Whitney *U* test was used. One-sided *P* values were chosen because the a priori hypothesis applied to the experiments was predicated upon results showing that the MAbs to the surface polysaccharides would only be interpreted to have a protective effect if administration reduced bacterial levels and tissue pathology compared to the irrelevant control MAb. Both the absence of an effect or enhancement of infection would be interpreted as a lack of efficacy of the MAb and indicate it was not a candidate for further therapeutic evaluations. Either an outcome showing no effect or enhancement of infection would lead to acceptance of the null hypothesis. The Prism statistical software (GraphPad, San Diego, CA, USA) was used and *P* values <0.05 were considered significant.

## Results

### Prophylactic and Therapeutic Efficacy of Antibody to PNAG in Murine Conjunctivitis

**Figure 1:**
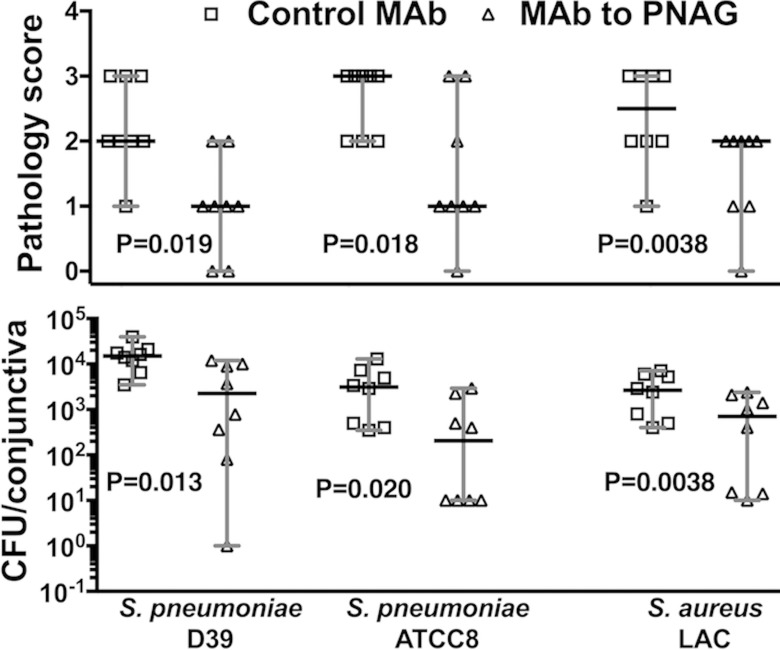
Intraperitoneal administration of MAb to PNAG preinfection and postinfection reduces bacterial burdens and conjunctival pathology due to S. pneumoniae (D-39 and ATCC8) and S. aureus (LAC) after 48 hours of infection in A/J mice. Eighteen hours prior to infection and 4, 24, and 32 hours postinfection, 200 μg of the human IgG1 monoclonal antibody MAb F598 to PNAG or control IgG1 MAb F429 were injected IP. Mice (n = 8 per group) were euthanized at 48 hours postinfection and pathology scores (upper row) and CFU/conjunctiva (lower row) were determined. Symbols represent individual animals, black lines the median, and gray lines the 95% CI, and one-sided P values were determined by nonparametric t-tests.

We initially tested the efficacy of prophylactic administration of antibody to PNAG in the setting of conjunctivitis by injecting either the MAb to PNAG or the isotype control antibody to alginate IP 18 hours preinfection and 4, 24, and 32 hours postinfection. Prophylactic injection is not only the most likely method to show an effect in a newly derived experimental infection, but it also mimics potential clinical use in the setting of high risk for recurrent infections or potential efficacy from active immunization. Two PNAG-positive *S. pneumoniae* strains, D-39 and ATCC8, and a PNAG-positive *S. aureus* MRSA USA300 strain, LAC, were used for infections. After 48 hours, we scored the conjunctivae for pathology and determined bacterial levels in the ocular tissues after euthanasia. Intraperitoneal injection of antibody to PNAG reduced the median conjunctival pathology as well as the median bacterial burdens 5- to 15-fold in the conjunctiva (Fig. 1) for both *S. pneumoniae* strains and the MRSA isolate.

### Therapeutic Efficacy of Monoclonal Antibody to PNAG in *S. pneumoniae* and *S. aureus* Conjunctivitis

**Figure 2:**
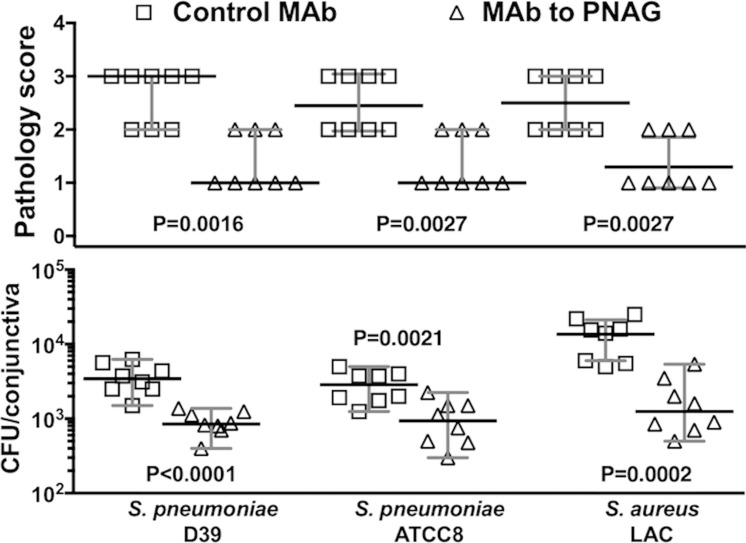
Postinfection intraconjunctival administration of MAb to PNAG reduces bacterial burdens and conjunctival pathology due to S. pneumoniae (strains D-39 and ATCC8) and S. aureus (strain LAC) after 48 hours of conjunctival infection in A/J mice. A total of 10 μg of control IgG MAb (□) or MAb to PNAG (▵) were injected into the conjunctiva 4, 24, and 32 hours postinfection. Mice (n = 8 per group) were euthanized at 48 hours postinfection and pathology scores (upper row) and CFU/conjunctiva (lower row) were determined. Symbols represent individual animals, black lines the median, and gray lines the 95% CI, and one-sided P values were determined by nonparametric t-tests.

We assessed the therapeutic efficacy of the MAb to PNAG in the conjunctivitis model by injecting it into conjunctivae 4, 24, and 32 hours after *S. pneumoniae* and *S. aureus* infection was established. Intraconjunctival injection would most likely lead to maximally achievable therapeutic levels in the infected tissue. We found that the intraconjunctival injection of the MAb to PNAG reduced the median tissue pathology and the median bacterial counts 3- to 11-fold for the *S. pneumoniae* and *S. aureus* strains when compared with the IgG control MAb (Fig. 2).

**Figure 3:**
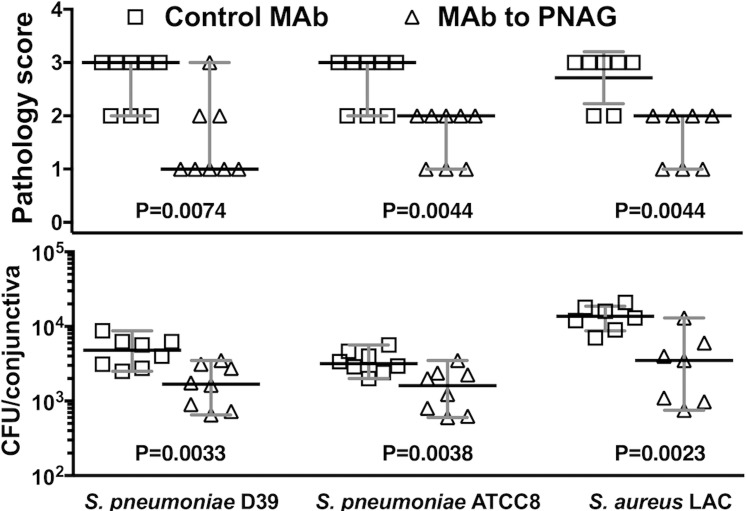
Postinfection topical administration of MAb to PNAG reduces bacterial burdens and conjunctival pathology due to S. pneumoniae (D-39 and ATCC8) and S. aureus (LAC) after 48 hours of infection in A/J mice. A total of 10 μg of control IgG MAb (□) or MAb to PNAG (▵) were administered topically onto conjunctiva 4, 24, and 32 hours postinfection. Mice (n = 7 to 8 per group) were euthanized at 48 hours post infection and pathology scores (upper row) and CFU/conjunctiva (lower row) determined. Symbols represent individual animals, black lines the median, and gray lines the 95% CI, and one-sided P values were determined by nonparametric t-tests.

We also determined the therapeutic efficacy of the MAb to PNAG by topical administration, a less invasive means of treatment, by applying 10 μg of MAb in 5 μL volumes onto the conjunctiva 4, 24, and 32 hours after *S. pneumoniae* or *S. aureus* infection. Topical administration lowered the median conjunctival pathology score and the median CFU/conjunctiva 3- to 4-fold (Fig. 3) in this model. Thus, multiple routes of administration, including systemic, intraconjunctival, or topical administration can deliver sufficient MAb to the conjunctiva to reduce bacterial burdens and limit inflammatory damage.

### Efficacy of Antibody to *P. aeruginosa* Alginate in Conjunctival Infection

**Figure 4:**
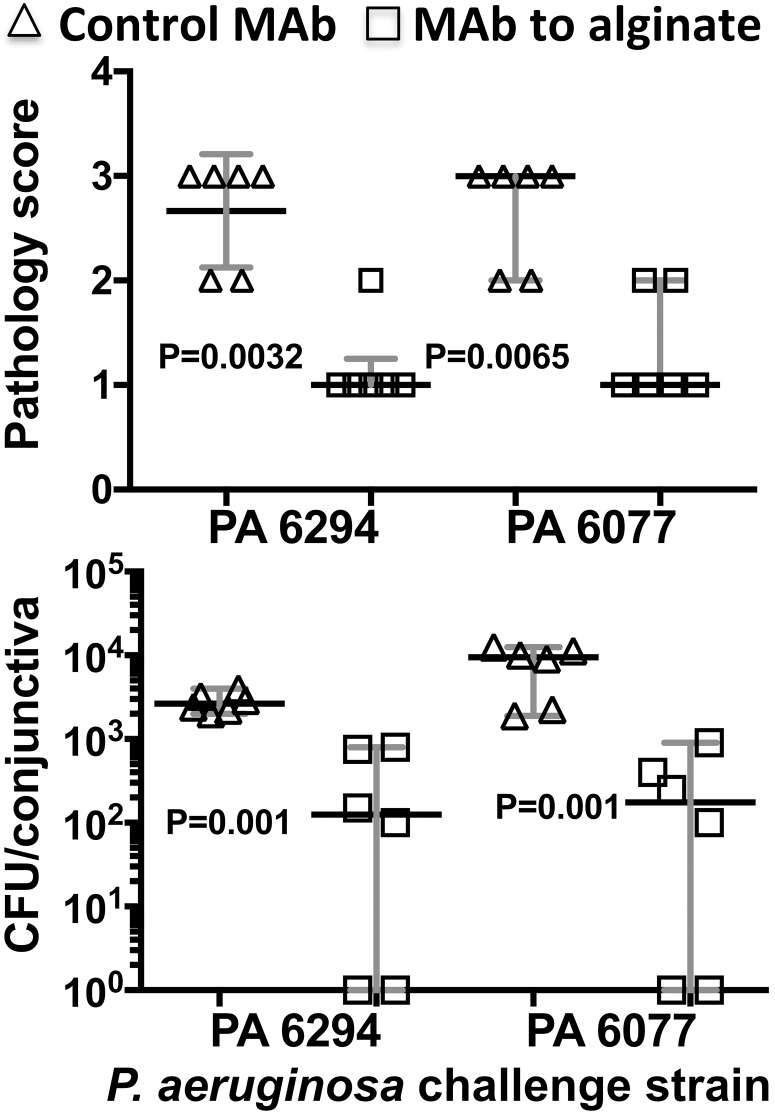
Postinfection administration of MAb to alginate reduces bacterial burdens and conjunctival pathology due to P. aeruginosa after 48 hours of conjunctival infection. A total of 10 μg of control IgG MAb to PNAG (▵) or anti-P. aeruginosa alginate MAb (□) were administered topically onto conjunctiva 4, 24, and 32 hours postinfection. A/J mice (n = 6 per group) were euthanized at 48 hours postinfection and pathology scores (A) and CFU/cornea (B) determined. Symbols represent individual animals, black lines the medians, and gray lines the 95% C.I., and P values were determined by nonparametric t-tests.

Because *P. aeruginosa* is an important cause of conjunctivitis in the elderly^10^ and can cause serious outbreaks in neonatal intensive care units,^12^ we tested the MAb to alginate for efficacy against this pathogen in the conjunctivitis model. This assay also validated the functionality of the anti-*P. aeruginosa* MAb in vivo when used against its cognate target. In comparison to the effects of the irrelevant anti-PNAG MAb F598 on *P. aeruginosa* conjunctivitis, MAb F429 to alginate effectively reduced corneal pathology and bacterial burdens due to two clinical isolates (Fig. 4), validating its protective activity in the setting of conjunctivitis.

### Need for Innate Lymphocytes for Protective Efficacy and Resistance to Infection

**Figure 5:**
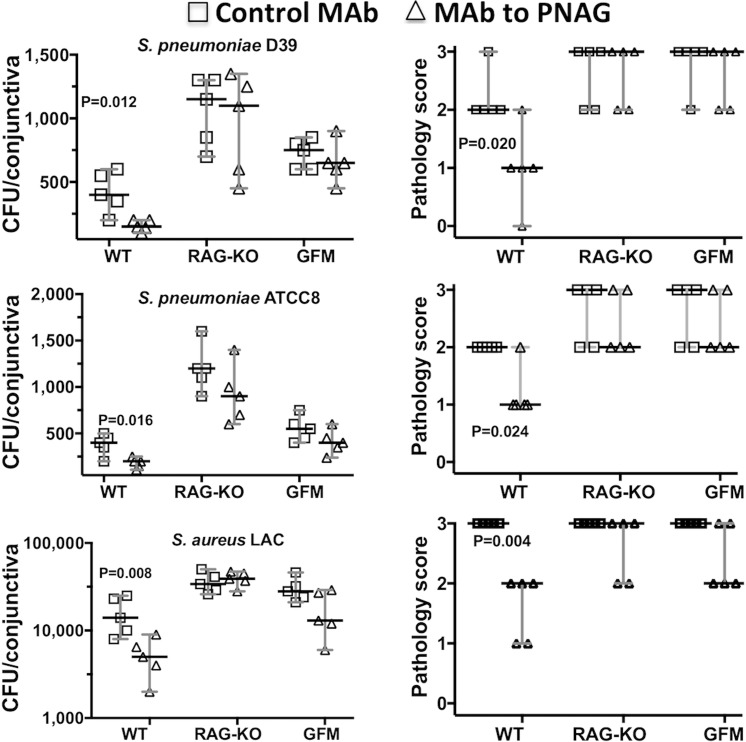
Postinfection administration of MAb to PNAG reduces bacterial burdens and conjunctival pathology due to S. pneumoniae (D-39 and ATCC8) or S. aureus (LAC) in WT C57Bl/6, but not in RAG 1 KO or germ-free mice (GFM) after 48 hours. A total of 10 μg of control IgG MAb (□) or MAb to PNAG (▵) were injected into the conjunctiva 4, 24, and 32 hours postinfection. Mice (n = 5 per group) were euthanized at 48 hours postinfection and pathology scores (upper row) and CFU/conjunctiva (lower row) were determined. Symbols represent individual animals, bars the median, and error bars the 95% CI, and one-sided P values were determined by nonparametric t-tests. None of the differences between MAb to PNAG or control MAb in RAG 1 KO or GFM mice were significant (P > 0.22).

Next, we measured the protective activity of the MAb to PNAG in conjunctival infections in WT C57Bl/6 mice, as they served as controls for analysis of immunity in RAG 1 KO and germ-free mice in this genetic background. We noted a 2- to 3-fold reduction in the median CFU in the conjunctiva for all bacterial strains tested in comparison to the microbial burdens in C57Bl/6 mice given the control MAb treatment (Fig. 5). However, the C57Bl/6 mice had notably lower *S. pneumoniae* burdens in the conjunctiva compared to the A/J mice (Figs. 1–3), indicative of a greater resistance of the C57Bl/6 mouse strain to *S. pneumoniae* conjunctivitis. In contrast, the *S. aureus* LAC strain was comparably virulent in the A/J and C57Bl/6 mice.

When the MAb to PNAG was injected into the conjunctivae of RAG 1 KO mice, deficient in all lymphocytes that undergo B- or T-cell receptor rearrangements, there was no reduction in pathology score or bacterial burdens for either *S. pneumoniae* (D-39 or ATCC8) or *S. aureus* LAC (Fig. 5) when compared to mice given the control IgG1 MAb.

Along the same lines, as germ-free mice are well known to lack fully mature immune systems,^30^ we evaluated whether the native microbiome affects the activity of immune cells at the conjunctival ocular site. As with the RAG 1 KO mice, germ-free mice treated with the MAb to PNAG did not have reduced bacterial levels or conjunctival pathology scores in comparison to mice receiving the control MAb (Fig. 5).

**Figure 6:**
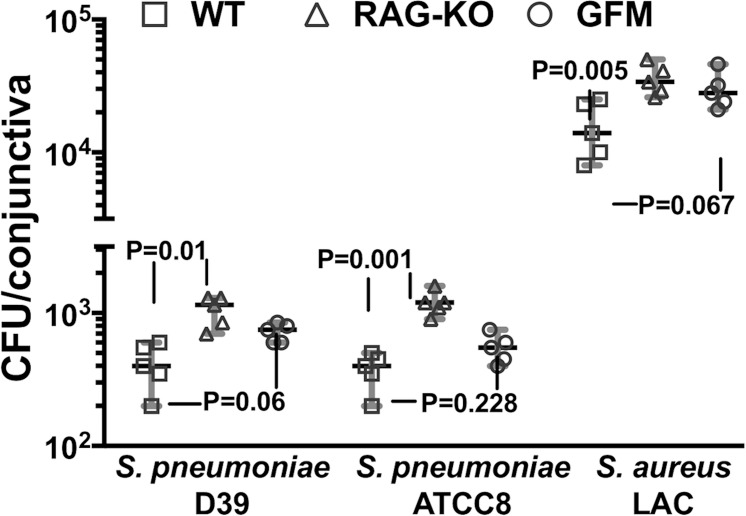
Comparison of the effect of a lack of lymphocytes in RAG-KO mice or an effect from the lack of a microbiota in germ-free mice on bacterial conjunctival levels after 48 hours of infection. Mice (n = 5 per group) given control MAb only were euthanized at 48 hours postinfection and CFU/conjunctiva was determined. Symbols represent individual animals, black lines the median, and gray lines the 95% CI, and P values were determined by nonparametric ANOVA. Overall ANOVA for analysis of all three organisms P < 0.008. P values depicted on figure are from pair-wise comparisons using the two-stage linear step-up procedure of Benjamini et al.^35^

To ascertain if the lack of adaptive lymphocytes in RAG 1 KO mice or a microbiome in germ-free mice impacted the bacterial burdens or pathology, we compared these factors in the WT, RAG 1 KO, or germ-free mice receiving only control MAb. We observed that the bacterial burdens were significantly elevated in the RAG 1 KO mice compared with WT C57Bl/6 mice for all three bacterial strains tested, although the differences in bacterial burdens between WT and germ-free mice were not significant at *P* ≤ 0.05 (Fig. 6). This suggests a need for T-cell receptor- or B-cell receptor-bearing lymphocytes in the innate immune control of bacterial conjunctivitis, whereas the lack of a microbiome had a minimal impact on basic virulence. There were no significant differences in the conjunctival pathology scores among the WT, RAG 1 KO, or germ-free mice given only the control MAb (Fig. 5), possibly reflective of the limitations of this model to generate more precise differences in pathology.

### Histopathologic and Myeloperoxidase Evaluations of Effects of Immunity to PNAG on Conjunctival Pathology

**Figure 7:**
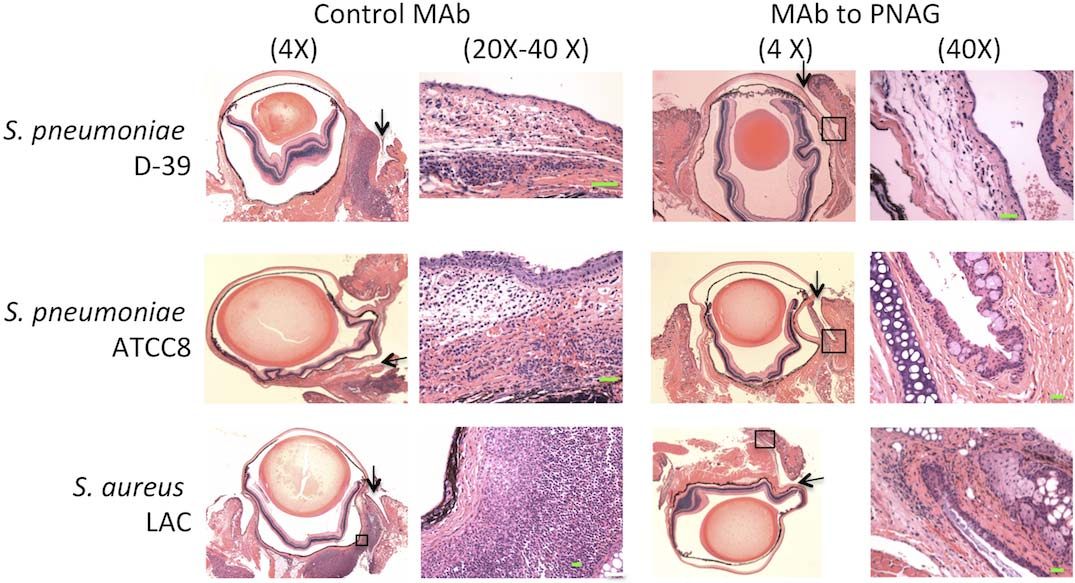
Postinfection administration of MAb to PNAG reduces conjunctival PMN infiltration due to S. pneumoniae (D-39 and ATCC8) and S. aureus (LAC) after 48 hours of conjunctival infection in A/J mice. A total of 10 μg of control IgG MAb or MAb to PNAG were injected into the conjunctiva 4, 24, and 32 hours postinfection in mice infected with S. pneumoniae D-39 or ATCC8 or S. aureus LAC. Control MAb-treated mice showed a large infiltrate of inflammatory cells and obvious edema in conjunctival areas, whereas mice treated with MAb to PNAG had only low levels of inflammatory cells and little edema. Arrows point to site of injection in the conjunctiva. Green bars: 10 μm. Boxed areas, when shown, indicated magnified area.

**Figure 8:**
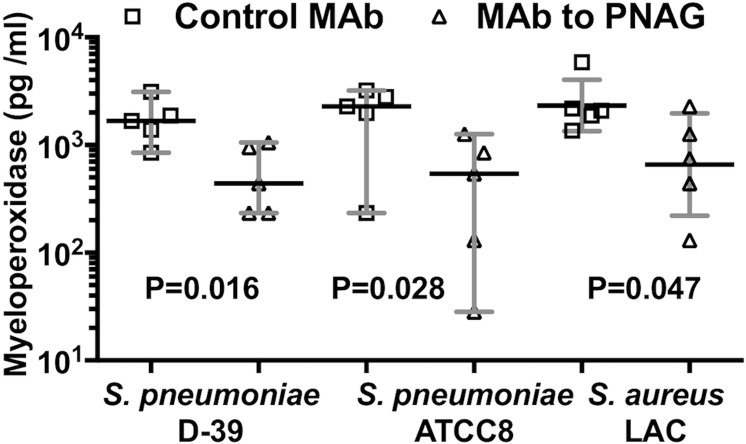
Postinfection administration of MAb to PNAG reduces conjunctival myeloperoxidase due to S. pneumoniae (D-39 and ATCC8) and S. aureus (LAC) after 48 hours of conjunctival infection in A/J mice. A total of 10 μg of control IgG MAb (□) or MAb to PNAG (▵) were injected into the conjunctiva 4, 24, and 32 hours postinfection with S. pneumoniae (D-39, ATCC8) or S. aureus LAC. Mice were euthanized at 48 hours postinfection and MPO/conjunctiva was determined. Symbols represent individual animals, black lines the medians, and gray lines the 95% CI, and P values were determined by one-sided nonparametric t-tests.

Histopathologic studies of conjunctiva following 48 hours of infection with *S. pneumoniae* (D-39 or ATCC8) or *S. aureus* LAC showed a large infiltrate of inflammatory cells in animals given the control MAb, whereas there was much less inflammation and edema in the conjunctiva of mice treated with MAb to PNAG (Figs. 7A–F). Consistent with these pathology findings, quantitative analysis of MPO levels in mouse corneas 48 hours postinfection showed significantly less MPO in tissues from animals given the anti-PNAG MAb, indicative of reduced PMN presence in these conjunctiva after 48 hours (Fig. 8).

## Discussion

We determined the protective efficacy in a murine model of conjunctivitis of a fully human IgG1 MAb to PNAG against *S. pneumoniae* or MRSA, as well as a MAb to *P. aeruginosa* alginate against this Gram-negative pathogen. We chose to evaluate local and systemic passive therapy in the setting of conjunctivitis, as these are not only the most likely clinical applications for MAb immunotherapies, but it is unlikely that a clinical test of preventative, active immunization would be undertaken in this setting due to the unduly large number of individuals that would be needed for such an evaluation. We found that the MAb to PNAG given by either an IP or intra-conjunctival injection, or applied topically, reduced conjunctival pathology and bacterial counts in these tissues. As PNAG production is commonly detected amongst many pathogens that cause eye infections,^15^ it is possible that passive administration of the human MAb to PNAG could provide an additional approach to treat many different microbial conjunctival infections.

We also tested a MAb to *P. aeruginosa* alginate^29^ in the conjunctivitis model to validate its efficacy and demonstrated its functional and protective capacity in this part of the ocular tissues. This MAb has been previously shown to mediate protective immunity to *P. aeruginosa* keratitis,^25,31^ and the evaluation here of its activity in the murine conjunctiva extends the potential utility of this reagent for use against *P. aeruginosa* bacterial conjunctivitis.

It is well established that antibody to surface polysaccharides of Gram-positive bacteria require complement and phagocytic cells, most often PMN, as cofactors to mediate killing and protective immunity. However, the need for additional cellular immune effectors is less well appreciated. We found that the MAb to PNAG was unable to reduce bacterial burdens and tissue pathology in the murine conjunctiva in both lymphocyte-deficient RAG 1 KO mice, and in germ-free mice lacking a microbiome-matured immune system. These findings are consistent with our previous report^22^ showing that the MAb to PNAG is only protective against *S. aureus* corneal infections when both lymphocytes and a microbiome-induced set of effectors are present. We also noted that in control mice challenged with Gram-positive pathogens, lymphocyte-deficient RAG 1 KO mice had higher bacterial levels in the conjunctiva 48 hours postinfection compared with WT C57l/6 mice. Notably, pathology scores did not differ, perhaps reflecting a limitation of the murine conjunctivitis model to detect smaller differences or the need for longer or more detailed observations regarding conjunctival pathology and its relationship to microbial burdens. Additionally, differences in bacterial burdens between WT and germ-free C567Bl/6 mice were not significant at *P* ≤ 0.05. Overall, we have established with these animal models an impact of lymphocyte effectors and microbiome-induced immunity to achieve maximal adaptive immunity in ocular tissues.

Both the MAb to PNAG and a vaccine composed of a synthetic oligosaccharide of pentameric glucosamine linked to a carrier protein, tetanus toxoid^15,32^, are in phase 1 or phase 2 human clinical trials (ClinicalTrials.gov identifier NCT03222401 for the MAb, NCT02853617 for the vaccine), as is the MAb to *P. aeruginosa* alginate (ClinicalTrials.gov identifier for phase 1 study, NCT02486770; for phase 2 study, NCT03027609). Thus, these reagents could be investigated in the near term for their efficacy in the setting of ocular infections, as infectious diseases are the fourth most common cause of preventable blindness in humans.^33^ The MAbs, in particular, could be useful for prophylaxis in a setting of high risk for ocular infection, such as following eye trauma or individuals with recurrent infections, or as adjunctive chemotherapy along with standards of care for therapeutic interventions in established infections. Systemic and topical administration of the MAb has been effective in mice with keratitis^15,23,34^ and, as shown here, conjunctivitis. Although the latter disease is much less sight-threatening, it is a costly disease to treat,^13^ thus providing justification for clinically useful, cost-effective interventions. Notably, topical and intraconjunctival injections of small amounts of MAb were successful in reducing conjunctival bacterial burdens and disease, which could make the local use of small amounts of a MAb highly cost-effective.

In conclusion, we have found that systemic administration of antibody to PNAG prior to infection provided protection against *S. pneumoniae* and *S. aureus* conjunctivitis. Similarly, therapeutic administration of intraconjunctival or topical antibody postinfection was efficacious, situations mimicking that appropriate for treatment of an already infected conjunctiva. Along with antibody, lymphocytes and microbiome-induced immune system maturation were also required for maximal lowering of *S. pneumoniae* and *S. aureus* bacterial burdens and pathology. These findings support human studies to actually determine the utility of immunotherapeutic prevention or treatment of major microbial causes of eye infections targeting the broadly-expressed surface antigen, PNAG, a process that is now facilitated by testing of appropriate reagents in human phase 1 and 2 trials.
